# Supplementary material for: MetaCAM as an ensemble-based class activation mapping framework improves model explainability
Source: Sci Rep. 2026 Mar 30;16:10613. doi: 10.1038/s41598-026-42879-0 (PMC13039914; doi:10.1038/s41598-026-42879-0)
Supplement: Supplementary file 1 — Supplementary Information. [file 41598_2026_42879_MOESM1_ESM.pdf]

Supplementary Material

MetaCAM as an Ensemble-Based Class Activation Mapping Framework Improves Model Explainability

Kevin Dick[1,3,7,+,*], Emily Kaczmarek[1,+], Olivier X. Miguel[2], Alexa C. Bowie[2], Robin Ducharme[2], Alysha L.J. Dingwall-Harvey[1,2,3], Steven Hawken[1,2,4,5], Christine M. Armour[1,3,6,7,8,9], and Mark C. Walker[1,2,3,4,6,8,9,10]

[1]Children's Hospital of Eastern Ontario Research Institute, Ottawa, Canada
[2]Clinical Epidemiology Program, Ottawa Hospital Research Institute, Ottawa, Canada
[3]BORN Ontario, Children's Hospital of Eastern Ontario, Ottawa, Canada
[4]School of Epidemiology and Public Health, University of Ottawa, Ottawa, Canada
[5]Institute of Clinical Evaluative Sciences, Toronto, Canada
[6]Department of Obstetrics and Gynecology, University of Ottawa, Ottawa, Canada
[7]Prenatal Screening Ontario, Better Outcomes Registry & Network, Ottawa, Canada
[8]Department of Obstetrics, Gynecology & Newborn Care, The Ottawa Hospital, Ottawa, Canada
[9]Department of Pediatrics, University of Ottawa, Ottawa, Canada
[10]International and Global Health Office, University of Ottawa, Ottawa, Canada
[*]kdick@bornontario.on.ca
[+]these authors contributed equally to this work

ABSTRACT

The following supplementary materials are provided in support of the MetaCAM main article. Herein, we provide the necessary details to run the open-sourced implementation of MetaCAM, the tabulated result following from extensive high-performance computing (HPC) experiments, and additional details related to particular behaviors observed throughout our experimentation. Adjoined to this document is a `.ipynb` notebook with a minimum viable working example of MetaCAM.

1 MetaCAM Source Code

The code for MetaCAM is provided as an adjoined `.ipynb` notebook. This implementation builds upon an existing publicly available python package, `grad-cam`. The first three cells are used to define the MetaCAM class and initialize all requisite settings, and the final cell can be used to run MetaCAM with selected parameters.

The overall class parameters include the given CNN model (*model*), target layer for CAMs (*target_layer*), CAM parameters (*use_cuda*, *reshape_transform*), and boolean parameters for scaling MetaCAM output between $[0, 1]$, saving ROAD performance scores for all CAMs generated, and returning a `dict` containing CAM outputs (*normalization*, *save_all_scores*, *return_cams*).

The individual MetaCAM call has parameters to define the input image as a tensor (*image_tensor*), a list of CAMs with which to compute the MetaCAM output (*cams*), the desired class output (*category*), the percentiles with which to compute the ROAD metric (*road_percentiles*), a list of weighting functions for MetaCAM (*weighted_functions*), the k highest activated pixel thresholds with which to calculate MetaCAM (*thresholds*), a boolean parameter to run all individual CAMs with adaptive thresholding (*individual_cams*), and an output directory and file name parameter for saving the CSV with all performance metrics (*output_directory*, *file_name*).

We have provided the code with predefined parameters. This includes saving all CAM scores and returning all CAMs (which are subsequently plotted as PNG images following the MetaCAM call), using all MetaCAM weighting functions, and thresholding MetaCAM between 15-45% of the highest activated pixels.

2 High-Performance Computing Results of MetaCAM Inclusion/Exclusion Experiments

Among the systematic ensemble-based experiments we performed, where all possible combinations of 2^6 CAM group inclusions/exclusions were considered, we obtained summary tables explicitly ranking MetaCAM performance across top-k thresholds and in comparison to other MetaCAM weighting factors. We provide a complete tabulation of these results in Table S1 for the image "catdog", with target class 281 (cat), and the pre-trained ResNet152 model[1,2]. The results are ordered

by decreasing maximum-achievable ROAD on a per-experiment basis. These tabulated results are the basis for the various quantified MetaCAM results in the figures within the main article.

Hereafter, we present a simplified version of such tables when considering varying images, target classes, and pre-trained model architectures. For example, Table S2 contains the summarized results and relative ranking for the same *catdog* image and ResNet152 model with the target class instead set to 242 for the *boxer* dog breed. Similarly, Table S3 contains the summarized results and relative ranking for the *seasnake* image with target class 65 and the ResNet152 model. Beyond animal-focused examples, Table S4 lists HPC results for an image containing a tie knot with class id 906 and the same ResNet152 model. Finally, we tabulate the MetaCAM results when the target class is mismatched to the contents of the image, effectively generating meaningless results (Table S5). Here, as a negative control, we see that the ROAD values are severely diminished and MetaCAM does not outperform any other individual methods and generates random-like results (Table S5).

Table S1. Comprehensive MetaCAM HPC Results for Catdog with Class 281 and ResNet152

Experiment ID	Included CAMs	Max ROAD	Threshold of Max ROAD	k=15	k=16	k=17	k=18	k=19	k=20	k=21	k=22	k=23	k=24	k=25	k=26	k=27	k=28	k=29	k=30	k=31	k=32	k=33	k=34	k=35	k=36	k=37	k=38	k=39	k=40	k=41	k=42	k=43	k=44	k=45	MetaCAM Weighted	MetaCAM Softmax	MetaCAM Exp	MetaCAM Min-Max	MetaCAM
000011_EF	LayerCAM_FullGrad	**0.347**	23	0.214	0.281	0.304	0.318	0.321	0.309	0.327	0.333	0.347	0.319	0.318	0.298	0.295	0.296	0.286	0.262	0.249	0.249	0.245	0.216	0.201	0.185	0.185	0.180	0.210	0.201	0.191	0.175	0.173	0.191	0.189	0.129	0.124	0.127	0.117	0.126
000101_DF	AblationCAM_ScoreCAM_FullGrad	**0.347**	23	0.238	0.249	0.297	0.303	0.322	0.320	0.327	0.345	0.347	0.332	0.318	0.309	0.294	0.275	0.279	0.286	0.256	0.241	0.222	0.242	0.237	0.224	0.240	0.216	0.167	0.177	0.179	0.180	0.182	0.198	0.184	0.174	0.188	0.185	0.192	0.180
001001_CF	XGradCAM_FullGrad	**0.342**	22	0.228	0.220	0.237	0.266	0.265	0.289	0.320	0.342	0.333	0.327	0.321	0.305	0.300	0.305	0.265	0.256	0.260	0.251	0.233	0.242	0.252	0.238	0.220	0.223	0.202	0.167	0.181	0.164	0.172	0.174	0.183	0.156	0.215	0.169	0.213	0.176
100001_AF	HiResCAM_GradCAMElementwise_FullGrad	**0.342**	22	0.228	0.220	0.237	0.266	0.265	0.289	0.320	0.342	0.333	0.327	0.321	0.305	0.300	0.305	0.265	0.256	0.260	0.251	0.233	0.242	0.252	0.238	0.220	0.223	0.202	0.167	0.181	0.164	0.172	0.174	0.183	0.156	0.215	0.169	0.213	0.176
000111_DEF	AblationCAM_ScoreCAM_LayerCAM_FullGrad	**0.338**	25	0.137	0.169	0.182	0.216	0.264	0.297	0.294	0.302	0.309	0.320	0.338	0.310	0.289	0.272	0.295	0.259	0.262	0.271	0.256	0.239	0.228	0.243	0.212	0.219	0.197	0.169	0.176	0.180	0.177	0.189	0.185	0.167	0.192	0.146	0.173	0.147
100011_AEF	HiResCAM_GradCAMElementwise_LayerCAM_ FullGrad	**0.314**	25	0.170	0.164	0.153	0.170	0.197	0.268	0.284	0.301	0.304	0.290	0.314	0.284	0.269	0.275	0.282	0.284	0.249	0.257	0.268	0.252	0.243	0.229	0.223	0.215	0.185	0.183	0.172	0.173	0.168	0.181	0.184	0.168	0.210	0.162	0.209	0.151
001011_CEF	XGradCAM_LayerCAM_FullGrad	**0.314**	25	0.170	0.164	0.153	0.170	0.197	0.268	0.284	0.301	0.304	0.290	0.314	0.284	0.269	0.275	0.282	0.284	0.249	0.257	0.268	0.252	0.243	0.229	0.223	0.215	0.185	0.183	0.172	0.173	0.168	0.181	0.184	0.168	0.210	0.162	0.209	0.151
100101_ADF	HiResCAM_GradCAMElementwise_AblationCAM_ ScoreCAM_FullGrad	**0.303**	26	0.174	0.161	0.176	0.173	0.221	0.259	0.278	0.287	0.302	0.287	0.285	0.303	0.279	0.277	0.272	0.267	0.284	0.267	0.259	0.275	0.245	0.244	0.223	0.208	0.186	0.168	0.177	0.156	0.159	0.162	0.169	0.158	0.200	0.148	0.210	0.163
001101_CDF	XGradCAM_AblationCAM_ScoreCAM_FullGrad	**0.303**	26	0.174	0.161	0.176	0.173	0.221	0.259	0.278	0.287	0.302	0.287	0.285	0.303	0.279	0.277	0.272	0.267	0.284	0.267	0.259	0.275	0.245	0.244	0.223	0.208	0.186	0.168	0.177	0.156	0.159	0.162	0.169	0.158	0.200	0.148	0.210	0.163
010001_BF	GradCAM_GradCAMPlusPlus_FullGrad	**0.297**	25	0.146	0.170	0.153	0.159	0.185	0.264	0.272	0.278	0.287	0.288	0.297	0.291	0.271	0.259	0.262	0.286	0.284	0.251	0.260	0.254	0.230	0.213	0.225	0.198	0.205	0.183	0.168	0.169	0.164	0.179	0.181	0.169	0.213	0.147	0.201	0.168
000001_F	FullGrad	**0.296**	15	0.296	0.278	0.260	0.242	0.237	0.252	0.247	0.281	0.276	0.253	0.226	0.220	0.201	0.183	0.198	0.187	0.185	0.194	0.193	0.184	0.179	0.157	0.158	0.144	0.150	0.161	0.178	0.170	0.183	0.177	0.190	0.109	0.108	0.110	0.000	0.111
010011_BEF	GradCAM_GradCAMPlusPlus_LayerCAM_FullGrad	**0.284**	24	0.235	0.219	0.162	0.157	0.161	0.248	0.238	0.247	0.260	0.284	0.276	0.261	0.261	0.263	0.244	0.262	0.272	0.266	0.259	0.239	0.254	0.231	0.210	0.208	0.185	0.177	0.165	0.171	0.168	0.172	0.176	0.127	0.214	0.142	0.224	0.134
101101_ACDF	HiResCAM_GradCAMElementwise_XGradCAM_ AblationCAM_ScoreCAM_FullGrad	**0.282**	25	0.206	0.206	0.163	0.162	0.149	0.238	0.256	0.241	0.248	0.263	0.282	0.249	0.255	0.241	0.242	0.275	0.269	0.269	0.270	0.274	0.247	0.241	0.215	0.208	0.202	0.189	0.175	0.184	0.181	0.165	0.164	0.180	0.214	0.155	0.225	0.149
001111_CDEF	XGradCAM_AblationCAM_ScoreCAM_LayerCAM_ FullGrad	**0.281**	25	0.234	0.187	0.168	0.162	0.168	0.244	0.245	0.272	0.263	0.279	0.281	0.260	0.272	0.270	0.252	0.255	0.276	0.255	0.269	0.255	0.251	0.216	0.233	0.200	0.180	0.169	0.165	0.155	0.158	0.164	0.165	0.145	0.202	0.163	0.230	0.165
100111_ADEF	HiResCAM_GradCAMElementwise_AblationCAM_ ScoreCAM_LayerCAM_FullGrad	**0.281**	25	0.234	0.187	0.168	0.162	0.168	0.244	0.245	0.272	0.263	0.279	0.281	0.260	0.272	0.270	0.252	0.255	0.276	0.255	0.269	0.255	0.251	0.216	0.233	0.200	0.180	0.169	0.165	0.155	0.158	0.164	0.165	0.145	0.203	0.164	0.230	0.164
010101_BDF	GradCAM_GradCAMPlusPlus_AblationCAM_ ScoreCAM_FullGrad	**0.281**	25	0.219	0.195	0.164	0.149	0.176	0.246	0.251	0.248	0.240	0.268	0.281	0.271	0.250	0.259	0.248	0.264	0.272	0.260	0.264	0.250	0.236	0.241	0.227	0.214	0.179	0.164	0.173	0.157	0.160	0.156	0.166	0.153	0.201	0.173	0.223	0.167
011101_BCDF	GradCAM_GradCAMPlusPlus_XGradCAM_ AblationCAM_ScoreCAM_FullGrad	**0.281**	34	0.230	0.198	0.187	0.194	0.174	0.232	0.249	0.256	0.234	0.240	0.243	0.260	0.249	0.233	0.239	0.254	0.266	0.251	0.261	0.281	0.242	0.246	0.220	0.212	0.182	0.187	0.178	0.169	0.160	0.162	0.166	0.163	0.218	0.147	0.227	0.156
110101_ABDF	HiResCAM_GradCAMElementwise_GradCAM_ GradCAMPlusPlus_AblationCAM_ScoreCAM_ FullGrad	**0.281**	34	0.230	0.198	0.187	0.194	0.174	0.232	0.249	0.256	0.234	0.240	0.243	0.260	0.249	0.233	0.239	0.254	0.266	0.251	0.261	0.281	0.242	0.246	0.220	0.212	0.182	0.187	0.178	0.169	0.160	0.162	0.166	0.160	0.210	0.147	0.220	0.154
101001_ACF	HiResCAM_GradCAMElementwise_XGradCAM_ FullGrad	**0.279**	31	0.191	0.173	0.153	0.142	0.187	0.247	0.271	0.276	0.258	0.277	0.270	0.256	0.272	0.258	0.254	0.268	0.279	0.270	0.259	0.255	0.246	0.227	0.231	0.194	0.206	0.175	0.178	0.176	0.151	0.169	0.170	0.156	0.215	0.156	0.216	0.156
111001_ABCF	HiResCAM_GradCAMElementwise_GradCAM_ GradCAMPlusPlus_XGradCAM_FullGrad	**0.276**	34	0.235	0.205	0.197	0.205	0.185	0.237	0.237	0.264	0.236	0.242	0.232	0.252	0.247	0.236	0.221	0.248	0.269	0.247	0.268	0.276	0.258	0.238	0.246	0.207	0.181	0.192	0.183	0.177	0.184	0.173	0.168	0.170	0.216	0.157	0.205	0.152
000010_E	LayerCAM	**0.276**	26	0.207	0.203	0.204	0.215	0.220	0.235	0.241	0.223	0.230	0.263	0.254	0.276	0.235	0.206	0.200	0.185	0.227	0.261	0.258	0.252	0.219	0.206	0.190	0.189	0.196	0.178	0.178	0.170	0.153	0.163	0.162	0.114	0.117	0.109	0.000	0.118
111011_ABCEF	HiResCAM_GradCAMElementwise_GradCAM_ GradCAMPlusPlus_XGradCAM_LayerCAM_ FullGrad	**0.275**	34	0.232	0.204	0.202	0.215	0.209	0.244	0.234	0.253	0.250	0.240	0.248	0.235	0.247	0.222	0.227	0.250	0.271	0.245	0.273	0.275	0.254	0.244	0.237	0.212	0.174	0.191	0.184	0.176	0.166	0.170	0.177	0.152	0.216	0.180	0.211	0.176
111111_ABCDEF	HiResCAM_GradCAMElementwise_GradCAM_ GradCAMPlusPlus_XGradCAM_AblationCAM_ ScoreCAM_LayerCAM_FullGrad	**0.274**	34	0.233	0.199	0.205	0.217	0.183	0.237	0.225	0.253	0.254	0.244	0.238	0.243	0.234	0.231	0.223	0.236	0.256	0.246	0.272	0.274	0.254	0.246	0.244	0.213	0.175	0.183	0.178	0.170	0.182	0.161	0.169	0.164	0.211	0.147	0.227	0.159
000110_DE	AblationCAM_ScoreCAM_LayerCAM	**0.274**	26	0.219	0.211	0.201	0.200	0.211	0.235	0.246	0.236	0.254	0.261	0.261	0.274	0.255	0.208	0.211	0.204	0.233	0.270	0.253	0.257	0.255	0.237	0.250	0.204	0.167	0.170	0.168	0.172	0.166	0.168	0.172	0.148	0.193	0.169	0.190	0.167
110110_ABDE	HiResCAM_GradCAMElementwise_GradCAM_ GradCAMPlusPlus_AblationCAM_ScoreCAM_ LayerCAM	**0.273**	33	0.199	0.224	0.199	0.222	0.230	0.238	0.233	0.251	0.234	0.236	0.262	0.255	0.237	0.225	0.188	0.196	0.211	0.229	0.273	0.262	0.259	0.261	0.234	0.207	0.198	0.182	0.177	0.188	0.187	0.177	0.155	0.172	0.205	0.166	0.222	0.158
011110_BCDE	GradCAM_GradCAMPlusPlus_XGradCAM_ AblationCAM_ScoreCAM_LayerCAM	**0.273**	33	0.199	0.224	0.199	0.222	0.230	0.238	0.233	0.251	0.234	0.236	0.262	0.255	0.237	0.225	0.188	0.196	0.211	0.229	0.273	0.262	0.259	0.261	0.234	0.207	0.198	0.182	0.177	0.188	0.187	0.177	0.155	0.171	0.215	0.166	0.224	0.154
011001_BCF	GradCAM_GradCAMPlusPlus_XGradCAM_ FullGrad	**0.273**	34	0.238	0.234	0.170	0.169	0.168	0.225	0.241	0.245	0.243	0.261	0.261	0.240	0.239	0.226	0.255	0.257	0.259	0.260	0.254	0.273	0.233	0.247	0.227	0.178	0.192	0.167	0.170	0.162	0.144	0.154	0.159	0.164	0.211	0.164	0.210	0.165
110001_ABF	HiResCAM_GradCAMElementwise_GradCAM_ GradCAMPlusPlus_FullGrad	**0.273**	34	0.238	0.234	0.170	0.169	0.168	0.225	0.241	0.245	0.243	0.261	0.261	0.240	0.239	0.226	0.255	0.257	0.259	0.260	0.254	0.273	0.233	0.247	0.227	0.178	0.192	0.167	0.170	0.162	0.144	0.154	0.159	0.162	0.211	0.164	0.208	0.165
000100_D	AblationCAM_ScoreCAM	**0.272**	24	0.233	0.220	0.175	0.185	0.213	0.206	0.261	0.237	0.246	0.272	0.258	0.255	0.245	0.217	0.205	0.214	0.222	0.230	0.252	0.233	0.240	0.249	0.225	0.208	0.195	0.173	0.180	0.175	0.187	0.169	0.157	0.194	0.194	0.192	0.000	0.194
110010_ABE	HiResCAM_GradCAMElementwise_GradCAM_ GradCAMPlusPlus_LayerCAM	**0.270**	34	0.184	0.218	0.191	0.226	0.224	0.202	0.220	0.242	0.243	0.229	0.250	0.235	0.223	0.224	0.181	0.191	0.214	0.227	0.252	0.270	0.252	0.245	0.228	0.203	0.179	0.171	0.166	0.185	0.167	0.165	0.152	0.159	0.208	0.158	0.210	0.172
011010_BCE	GradCAM_GradCAMPlusPlus_XGradCAM_ LayerCAM	**0.270**	34	0.184	0.218	0.191	0.226	0.224	0.202	0.220	0.242	0.243	0.229	0.250	0.235	0.223	0.224	0.181	0.191	0.214	0.227	0.252	0.270	0.252	0.245	0.228	0.203	0.179	0.171	0.166	0.185	0.167	0.165	0.152	0.160	0.217	0.154	0.209	0.174
101111_ACDEF	HiResCAM_GradCAMElementwise_XGradCAM_ AblationCAM_ScoreCAM_LayerCAM_FullGrad	**0.270**	34	0.216	0.198	0.189	0.194	0.178	0.223	0.259	0.252	0.224	0.239	0.255	0.260	0.254	0.226	0.240	0.259	0.255	0.245	0.251	0.270	0.241	0.241	0.214	0.196	0.192	0.181	0.172	0.176	0.156	0.159	0.162	0.157	0.215	0.153	0.219	0.162
101010_ACE	HiResCAM_GradCAMElementwise_XGradCAM_ LayerCAM	**0.270**	34	0.197	0.221	0.192	0.219	0.238	0.207	0.221	0.244	0.247	0.222	0.225	0.227	0.225	0.212	0.191	0.184	0.210	0.228	0.256	0.270	0.239	0.244	0.231	0.207	0.207	0.195	0.184	0.192	0.183	0.168	0.156	0.164	0.213	0.157	0.211	0.164
101011_ACEF	HiResCAM_GradCAMElementwise_XGradCAM_ LayerCAM_FullGrad	**0.270**	25	0.235	0.229	0.162	0.187	0.177	0.223	0.254	0.252	0.247	0.260	0.270	0.255	0.234	0.245	0.257	0.255	0.259	0.257	0.267	0.261	0.234	0.253	0.232	0.206	0.181	0.169	0.174	0.156	0.151	0.151	0.167	0.165	0.214	0.171	0.219	0.159
111101_ABCDF	HiResCAM_GradCAMElementwise_GradCAM_ GradCAMPlusPlus_XGradCAM_AblationCAM_ ScoreCAM_FullGrad	**0.270**	35	0.228	0.208	0.187	0.215	0.204	0.232	0.236	0.254	0.245	0.228	0.238	0.234	0.243	0.236	0.230	0.249	0.254	0.244	0.267	0.268	0.270	0.244	0.245	0.188	0.183	0.196	0.185	0.183	0.188	0.172	0.168	0.176	0.212	0.146	0.219	0.154
001010_CE	XGradCAM_LayerCAM	**0.269**	34	0.182	0.227	0.198	0.224	0.235	0.229	0.235	0.254	0.243	0.243	0.259	0.248	0.226	0.233	0.188	0.193	0.217	0.232	0.256	0.269	0.254	0.251	0.232	0.220	0.177	0.167	0.171	0.184	0.172	0.170	0.155	0.158	0.213	0.171	0.210	0.167
100010_AE	HiResCAM_GradCAMElementwise_LayerCAM	**0.269**	34	0.182	0.227	0.198	0.224	0.235	0.229	0.235	0.254	0.243	0.243	0.259	0.248	0.226	0.233	0.188	0.193	0.217	0.232	0.256	0.269	0.254	0.251	0.232	0.220	0.177	0.167	0.171	0.184	0.172	0.170	0.155	0.158	0.213	0.171	0.210	0.167
101000_AC	HiResCAM_GradCAMElementwise_XGradCAM	**0.269**	35	0.205	0.214	0.201	0.203	0.203	0.199	0.211	0.243	0.251	0.241	0.224	0.217	0.227	0.213	0.207	0.179	0.194	0.227	0.227	0.257	0.269	0.225	0.228	0.242	0.228	0.218	0.211	0.201	0.195	0.189	0.192	0.218	0.219	0.215	0.213	0.214
101100_ACD	HiResCAM_GradCAMElementwise_XGradCAM_ AblationCAM_ScoreCAM	**0.269**	34	0.204	0.223	0.220	0.187	0.241	0.209	0.231	0.247	0.246	0.238	0.249	0.226	0.222	0.228	0.187	0.185	0.216	0.224	0.242	0.269	0.262	0.247	0.241	0.232	0.226	0.226	0.228	0.204	0.191	0.183	0.185	0.219	0.218	0.223	0.215	0.227
001110_CDE	XGradCAM_AblationCAM_ScoreCAM_LayerCAM	**0.268**	25	0.221	0.219	0.209	0.211	0.230	0.227	0.246	0.241	0.240	0.238	0.268	0.255	0.246	0.221	0.177	0.213	0.211	0.226	0.263	0.257	0.259	0.250	0.224	0.212	0.185	0.179	0.173	0.194	0.176	0.180	0.149	0.174	0.199	0.168	0.215	0.158
100110_ADE	HiResCAM_GradCAMElementwise_AblationCAM_ ScoreCAM_LayerCAM	**0.268**	25	0.221	0.219	0.209	0.211	0.230	0.227	0.246	0.241	0.240	0.238	0.268	0.255	0.246	0.221	0.177	0.213	0.211	0.226	0.263	0.257	0.259	0.250	0.224	0.212	0.185	0.179	0.173	0.194	0.176	0.180	0.149	0.174	0.199	0.168	0.215	0.158
001000_C	XGradCAM	**0.267**	35	0.209	0.216	0.200	0.204	0.208	0.202	0.212	0.246	0.249	0.235	0.223	0.219	0.225	0.216	0.207	0.182	0.193	0.227	0.226	0.257	0.267	0.227	0.229	0.248	0.225	0.213	0.210	0.201	0.190	0.190	0.192	0.215	0.218	0.219	0.000	0.213
100000_A	HiResCAM_GradCAMElementwise	**0.267**	35	0.209	0.216	0.200	0.204	0.208	0.202	0.212	0.246	0.249	0.235	0.223	0.219	0.225	0.216	0.207	0.182	0.193	0.227	0.226	0.257	0.267	0.227	0.229	0.248	0.225	0.213	0.210	0.201	0.190	0.190	0.192	0.215	0.218	0.219	0.000	0.213
100100_AD	HiResCAM_GradCAMElementwise_AblationCAM_ ScoreCAM	**0.267**	34	0.226	0.229	0.227	0.181	0.235	0.207	0.221	0.247	0.240	0.236	0.250	0.246	0.219	0.223	0.177	0.203	0.221	0.226	0.238	0.267	0.246	0.239	0.235	0.221	0.210	0.215	0.218	0.202	0.189	0.186	0.176	0.206	0.201	0.210	0.215	0.211
001100_CD	XGradCAM_AblationCAM_ScoreCAM	**0.267**	34	0.226	0.229	0.227	0.181	0.235	0.207	0.221	0.247	0.240	0.236	0.250	0.246	0.219	0.223	0.177	0.203	0.221	0.226	0.238	0.267	0.246	0.239	0.235	0.221	0.210	0.215	0.218	0.202	0.189	0.186	0.176	0.206	0.199	0.210	0.215	0.211
010100_BD	GradCAM_GradCAMPlusPlus_AblationCAM_ ScoreCAM	**0.267**	33	0.209	0.216	0.202	0.213	0.235	0.214	0.221	0.239	0.232	0.242	0.259	0.248	0.224	0.222	0.180	0.205	0.202	0.217	0.267	0.247	0.245	0.257	0.232	0.204	0.202	0.184	0.182	0.186	0.184	0.170	0.162	0.171	0.196	0.165	0.215	0.166
010000_B	GradCAM_GradCAMPlusPlus	**0.265**	34	0.188	0.219	0.202	0.227	0.223	0.191	0.216	0.243	0.239	0.225	0.231	0.223	0.231	0.212	0.194	0.183	0.211	0.214	0.255	0.265	0.239	0.251	0.226	0.195	0.202	0.182	0.177	0.186	0.182	0.170	0.160	0.160	0.212	0.148	0.221	0.149
111010_ABCE	HiResCAM_GradCAMElementwise_GradCAM_ GradCAMPlusPlus_XGradCAM_LayerCAM	**0.265**	34	0.192	0.218	0.195	0.223	0.223	0.194	0.219	0.245	0.240	0.224	0.231	0.230	0.224	0.210	0.187	0.181	0.211	0.223	0.263	0.265	0.242	0.241	0.232	0.205	0.214	0.192	0.181	0.189	0.175	0.164	0.166	0.168	0.210	0.157	0.206	0.159
101110_ACDE	HiResCAM_GradCAMElementwise_XGradCAM_ AblationCAM_ScoreCAM_LayerCAM	**0.264**	34	0.206	0.217	0.207	0.208	0.235	0.217	0.233	0.258	0.234	0.236	0.260	0.243	0.225	0.237	0.181	0.201	0.211	0.219	0.257	0.264	0.247	0.250	0.238	0.211	0.218	0.200	0.191	0.193	0.188	0.167	0.161	0.176	0.215	0.169	0.221	0.176
110011_ABEF	HiResCAM_GradCAMElementwise_GradCAM_ GradCAMPlusPlus_LayerCAM_FullGrad	**0.264**	34	0.224	0.182	0.190	0.201	0.199	0.221	0.244	0.245	0.233	0.249	0.254	0.261	0.240	0.218	0.244	0.262	0.256	0.255	0.256	0.264	0.239	0.244	0.217	0.176	0.179	0.165	0.165	0.152	0.153	0.155	0.166	0.171	0.210	0.161	0.205	0.156
011011_BCEF	GradCAM_GradCAMPlusPlus_XGradCAM_ LayerCAM_FullGrad	**0.264**	34	0.224	0.182	0.190	0.201	0.199	0.221	0.244	0.245	0.233	0.249	0.254	0.261	0.240	0.218	0.244	0.262	0.256	0.255	0.256	0.264	0.239	0.244	0.217	0.176	0.179	0.165	0.165	0.152	0.153	0.155	0.166	0.170	0.210	0.161	0.201	0.156
010110_BDE	GradCAM_GradCAMPlusPlus_AblationCAM_ ScoreCAM_LayerCAM	**0.263**	25	0.226	0.222	0.202	0.217	0.225	0.235	0.233	0.241	0.236	0.240	0.263	0.257	0.233	0.221	0.181	0.205	0.219	0.247	0.257	0.253	0.251	0.258	0.235	0.227	0.178	0.168	0.177	0.181	0.169	0.176	0.164	0.163	0.196	0.160	0.211	0.167
011100_BCD	GradCAM_GradCAMPlusPlus_XGradCAM_ AblationCAM_ScoreCAM	**0.263**	33	0.203	0.218	0.198	0.220	0.250	0.221	0.231	0.255	0.234	0.231	0.247	0.242	0.221	0.234	0.203	0.204	0.217	0.222	0.263	0.255	0.249	0.243	0.237	0.225	0.222	0.197	0.198	0.197	0.193	0.176	0.170	0.208	0.213	0.164	0.224	0.170
110100_ABD	HiResCAM_GradCAMElementwise_GradCAM_ GradCAMPlusPlus_AblationCAM_ScoreCAM	**0.263**	33	0.203	0.218	0.198	0.220	0.250	0.221	0.231	0.255	0.234	0.231	0.247	0.242	0.221	0.234	0.203	0.204	0.217	0.222	0.263	0.255	0.249	0.243	0.237	0.225	0.222	0.197	0.198	0.197	0.193	0.176	0.170	0.203	0.210	0.165	0.225	0.170
111110_ABCDE	HiResCAM_GradCAMElementwise_GradCAM_ GradCAMPlusPlus_XGradCAM_AblationCAM_ ScoreCAM_LayerCAM	**0.262**	34	0.194	0.223	0.199	0.217	0.234	0.208	0.244	0.242	0.238	0.219	0.250	0.237	0.216	0.228	0.182	0.194	0.220	0.218	0.258	0.262	0.246	0.252	0.232	0.206	0.220	0.195	0.189	0.185	0.183	0.164	0.162	0.175	0.212	0.164	0.213	0.162
010010_BE	GradCAM_GradCAMPlusPlus_LayerCAM	**0.260**	25	0.199	0.202	0.198	0.227	0.211	0.214	0.222	0.240	0.226	0.232	0.260	0.233	0.225	0.218	0.176	0.188	0.206	0.236	0.259	0.251	0.251	0.243	0.213	0.197	0.154	0.167	0.163	0.163	0.172	0.156	0.164	0.174	0.209	0.169	0.210	0.161
011000_BC	GradCAM_GradCAMPlusPlus_XGradCAM	**0.260**	34	0.201	0.217	0.200	0.227	0.229	0.181	0.216	0.247	0.246	0.219	0.226	0.217	0.213	0.209	0.189	0.176	0.201	0.206	0.235	0.260	0.240	0.234	0.230	0.214	0.200	0.192	0.188	0.189	0.180	0.161	0.162	0.169	0.215	0.164	0.212	0.162
110000_AB	HiResCAM_GradCAMElementwise_GradCAM_ GradCAMPlusPlus	**0.260**	34	0.201	0.217	0.200	0.227	0.229	0.181	0.216	0.247	0.246	0.219	0.226	0.217	0.213	0.209	0.189	0.176	0.201	0.206	0.235	0.260	0.240	0.234	0.230	0.214	0.200	0.192	0.188	0.189	0.180	0.161	0.162	0.171	0.213	0.169	0.214	0.161
011111_BCDEF	GradCAM_GradCAMPlusPlus_XGradCAM_ AblationCAM_ScoreCAM_LayerCAM_FullGrad	**0.259**	34	0.220	0.209	0.205	0.203	0.201	0.201	0.229	0.234	0.224	0.217	0.231	0.242	0.244	0.222	0.231	0.242	0.258	0.241	0.251	0.259	0.241	0.236	0.202	0.189	0.168	0.167	0.159	0.165	0.145	0.152	0.152	0.152	0.215	0.166	0.223	0.161
110111_ABDEF	HiResCAM_GradCAMElementwise_GradCAM_ GradCAMPlusPlus_AblationCAM_ScoreCAM_ LayerCAM_FullGrad	**0.259**	34	0.220	0.209	0.205	0.203	0.201	0.201	0.229	0.234	0.224	0.217	0.231	0.242	0.244	0.222	0.231	0.242	0.258	0.241	0.251	0.259	0.241	0.236	0.202	0.189	0.168	0.167	0.159	0.165	0.145	0.152	0.152	0.151	0.215	0.169	0.227	0.163
010111_BDEF	GradCAM_GradCAMPlusPlus_AblationCAM_ ScoreCAM_LayerCAM_FullGrad	**0.259**	25	0.233	0.218	0.183	0.177	0.157	0.207	0.243	0.240	0.227	0.229	0.259	0.256	0.241	0.230	0.232	0.258	0.255	0.252	0.254	0.238	0.237	0.212	0.224	0.180	0.173	0.152	0.161	0.139	0.146	0.146	0.164	0.164	0.199	0.152	0.224	0.150
111000_ABC	HiResCAM_GradCAMElementwise_GradCAM_ GradCAMPlusPlus_XGradCAM	**0.256**	34	0.192	0.225	0.201	0.222	0.212	0.175	0.210	0.252	0.243	0.212	0.220	0.217	0.219	0.205	0.190	0.171	0.195	0.205	0.231	0.256	0.247	0.232	0.231	0.214	0.204	0.207	0.207	0.187	0.177	0.167	0.168	0.221	0.211	0.170	0.211	0.160
111100_ABCD	HiResCAM_GradCAMElementwise_GradCAM_ GradCAMPlusPlus_XGradCAM_AblationCAM_ ScoreCAM	**0.254**	34	0.199	0.229	0.212	0.213	0.252	0.204	0.226	0.235	0.238	0.217	0.240	0.227	0.225	0.211	0.187	0.182	0.203	0.216	0.236	0.254	0.245	0.238	0.236	0.213	0.205	0.206	0.206	0.203	0.183	0.166	0.169	0.191	0.214	0.170	0.216	0.171

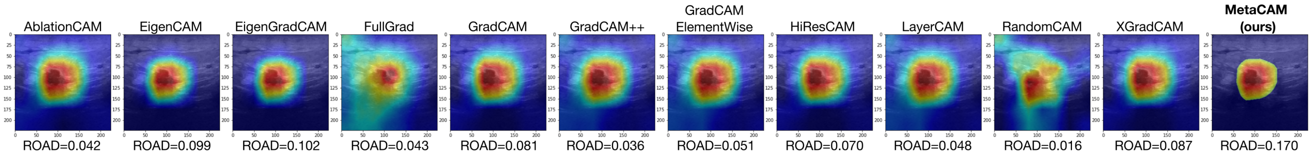

Figure S1. Comparison of individual CAMs using a fine-trained ResNet50 on normal and malignant breast cancer ultrasound images.

3 Rank-Order Summary of HPC Results

The HPC experiments generate relative rankings for a given image, target class, and pre-trained model. There exists extensive literature on comparing relative rankings of large-scale experiments. This work draws inspiration from recent work in the field of bioinformatics and therapeutic discovery, particularly as it pertains to normalizing data locally or globally[3–5]. Within the main article, we demonstrate that the rank-order distribution of maximum thresholds on an intra-experimental basis varies dramatically in morphology and suggests potential research directions that might exploit such rank-order distributions to further refine MetaCAM visual explanations.

4 Demonstration of MetaCAM in a Medical Image Application

Figure S1 demonstrates the effectiveness of MetaCAM in the high-criticality field of medical imaging (*e.g.*[6]). Here, we use a ResNet50 model[2] pre-trained on the ImageNet-1K dataset[1] and fine-tuned on a dataset of 133 normal and 210 malignant breast tissue ultrasound images[7] (image preprocessing modelled after[8]). Following training, a correctly classified malignant test sample is used to evaluate all CAM models. The adaptive thresholding and consensus-based formulation of MetaCAM achieves the highest ROAD performance, and refines the tumour area in the image, lending to improved visual explanation of model output. Consequently, we demonstrate that MetaCAM can improve performance and model interpretability in high-criticality fields such as medical imaging to better explain model predictions to end-users unfamiliar with AI.

5 Comparison of MetaCAM output to other CAMs

We have expanded the visual comparison of MetaCAM with other CAM methods to include an additional representative image category beyond those depicted in the main text (Figure S2).

6 Effect of Normalization on MetaCAM

We next evaluate the effect of normalization on MetaCAM ROAD performance. First, we examine the difference in MetaCAM performance when normalizing the summed pixel activations by the number of CAMs used to generate MetaCAM. Next, we evaluate if scaling the output between [0,1] changes the ROAD performance. As seen in Figure S3, there is negligible difference in performance when normalizing or scaling the summed pixel activations.

In the MetaCAM code, users are given the option to output the raw MetaCAM values (not scaled between [0,1]). However, MetaCAM formulation may output very small or negative values, particularly when using some of the weighted average MetaCAM options. These images may not appear as anticipated; for example, see Figure S4, which shows no heat map (right) or abnormal visualizations (left - due to the gradient attempting to find differences in extremely small raw MetaCAM values). For this reason, we recommend scaling the final output between [0,1], which is standard practice for individual CAMs.

References

1. Deng, J. *et al.* Imagenet: A large-scale hierarchical image database. In *2009 IEEE conference on computer vision and pattern recognition*, 248–255 (Ieee, 2009).

2. He, K., Zhang, X., Ren, S. & Sun, J. Deep residual learning for image recognition. In *Proceedings of the IEEE conference on computer vision and pattern recognition*, 770–778 (2016).

3. Dick, K. & Green, J. R. Reciprocal perspective for improved protein-protein interaction prediction. *Sci. reports* **8**, 1–12 (2018).

4. Dick, K. *et al.* Reciprocal perspective as a super learner improves drug-target interaction prediction (musdti). *Sci. Reports* **12**, 13237 (2022).

5. Dick, K., Kyrollos, D. G. & Green, J. R. Generalized reciprocal perspective. *arXiv preprint arXiv:2210.11616* (2022).

Table S2. MetaCAM HPC Results for Catdog with Class 242 and ResNet152

Experiment ID	Max ROAD	Thresh. Max ROAD	MetaCAM Weighted	MetaCAM Softmax	MetaCAM Exp	MetaCAM Min-Max	MetaCAM
000001_F	**0.372**	16	0.043	0.044	0.043	0.000	0.046
000010_E	**0.303**	15	0.052	0.054	0.054	0.000	0.053
011110_BCDE	**0.301**	15	0.039	0.050	0.036	0.042	0.034
110110_ABDE	**0.301**	15	0.040	0.052	0.036	0.044	0.037
100000_A	**0.296**	16	0.040	0.039	0.037	0.000	0.039
001000_C	**0.296**	16	0.040	0.039	0.037	0.000	0.039
010100_BD	**0.296**	15	0.037	0.055	0.034	0.047	0.036
010001_BF	**0.296**	15	0.062	0.053	0.057	0.049	0.058
101000_AC	**0.292**	16	0.039	0.037	0.038	0.037	0.037
100010_AE	**0.291**	15	0.041	0.052	0.039	0.054	0.039
001010_CE	**0.291**	15	0.041	0.052	0.039	0.054	0.039
011000_BC	**0.286**	15	0.041	0.057	0.034	0.051	0.035
110000_AB	**0.286**	15	0.037	0.057	0.034	0.050	0.035
110100_ABD	**0.286**	15	0.039	0.056	0.036	0.041	0.037
011100_BCD	**0.286**	15	0.039	0.053	0.036	0.044	0.040
010000_B	**0.286**	15	0.040	0.054	0.039	0.052	0.040
111010_ABCE	**0.281**	15	0.038	0.052	0.042	0.055	0.040
001100_CD	**0.281**	15	0.031	0.038	0.027	0.039	0.028
100100_AD	**0.281**	15	0.031	0.038	0.027	0.039	0.028
010010_BE	**0.280**	15	0.045	0.054	0.041	0.052	0.040
101010_ACE	**0.279**	16	0.038	0.054	0.038	0.055	0.037
110010_ABE	**0.278**	15	0.044	0.051	0.038	0.052	0.037
011010_BCE	**0.278**	15	0.045	0.052	0.035	0.050	0.036
000110_DE	**0.278**	15	0.036	0.052	0.038	0.053	0.036
111100_ABCD	**0.278**	15	0.035	0.054	0.036	0.038	0.035
111000_ABC	**0.277**	16	0.040	0.054	0.038	0.043	0.038
001110_CDE	**0.276**	15	0.043	0.052	0.034	0.043	0.037
100110_ADE	**0.276**	15	0.043	0.052	0.034	0.043	0.037
001011_CEF	**0.276**	15	0.064	0.052	0.066	0.060	0.066
100011_AEF	**0.276**	15	0.064	0.052	0.066	0.060	0.066
000011_EF	**0.276**	19	0.066	0.051	0.059	0.055	0.056
101110_ACDE	**0.275**	15	0.037	0.052	0.039	0.038	0.039
101100_ACD	**0.274**	15	0.035	0.038	0.031	0.037	0.032
010011_BEF	**0.272**	15	0.054	0.052	0.053	0.055	0.060
111110_ABCDE	**0.272**	16	0.036	0.052	0.037	0.039	0.039
111111_ABCDEF	**0.271**	16	0.041	0.051	0.043	0.057	0.040
110101_ABDF	**0.271**	16	0.038	0.056	0.037	0.053	0.035
011101_BCDF	**0.271**	16	0.037	0.056	0.035	0.057	0.036
111001_ABCF	**0.269**	15	0.038	0.055	0.037	0.054	0.035
010110_BDE	**0.269**	15	0.038	0.052	0.041	0.049	0.041
010111_BDEF	**0.267**	15	0.055	0.051	0.050	0.055	0.050
001101_CDF	**0.266**	15	0.055	0.041	0.039	0.068	0.039
100101_ADF	**0.266**	15	0.055	0.041	0.039	0.068	0.039
011111_BCDEF	**0.264**	15	0.040	0.051	0.040	0.053	0.039
110111_ABDEF	**0.264**	15	0.040	0.052	0.041	0.055	0.041
001001_CF	**0.264**	15	0.056	0.044	0.066	0.044	0.067
100001_AF	**0.264**	15	0.056	0.044	0.066	0.044	0.067
001111_CDEF	**0.260**	15	0.065	0.051	0.049	0.056	0.049
100111_ADEF	**0.260**	15	0.065	0.051	0.049	0.056	0.049
110011_ABEF	**0.260**	16	0.054	0.053	0.040	0.058	0.040
011011_BCEF	**0.260**	16	0.054	0.053	0.044	0.059	0.039
101101_ACDF	**0.257**	16	0.033	0.042	0.031	0.068	0.029
111011_ABCEF	**0.255**	15	0.042	0.053	0.039	0.060	0.038
000101_DF	**0.253**	15	0.067	0.045	0.071	0.044	0.065
111101_ABCDF	**0.252**	15	0.037	0.054	0.039	0.044	0.037
101011_ACEF	**0.251**	16	0.048	0.054	0.040	0.061	0.041
011001_BCF	**0.249**	15	0.042	0.055	0.039	0.054	0.038
110001_ABF	**0.249**	15	0.044	0.055	0.039	0.053	0.036
010101_BDF	**0.247**	16	0.053	0.053	0.042	0.061	0.047
101111_ACDEF	**0.247**	16	0.038	0.053	0.035	0.056	0.037
000111_DEF	**0.246**	15	0.058	0.052	0.054	0.073	0.056
000100_D	**0.245**	15	0.033	0.032	0.032	0.000	0.032
101001_ACF	**0.243**	15	0.040	0.043	0.036	0.033	0.037

Table S3. MetaCAM HPC Results for Seasnake with Class 65 and ResNet152

Experiment ID	Max ROAD	Thresh. Max ROAD	MetaCAM Weighted	MetaCAM Softmax	MetaCAM Exp	MetaCAM Min-Max	MetaCAM
000101_DF	**0.0462**	42	0.0587	0.0763	0.0733	0.0720	0.0748
000011_EF	**0.0456**	43	0.0751	0.0758	0.0796	0.0739	0.0781
000001_F	**0.0427**	45	0.0754	0.0753	0.0758	0.0000	0.0758
001001_CF	**0.0419**	43	0.0838	0.0735	0.0713	0.0743	0.0706
100001_AF	**0.0419**	43	0.0838	0.0735	0.0713	0.0743	0.0706
111001_ABCF	**0.0332**	41	0.0729	0.0747	0.0696	0.0679	0.0682
100011_AEF	**0.0301**	43	0.0749	0.0753	0.0555	0.0832	0.0625
000111_DEF	**0.0294**	40	0.0764	0.0757	0.0591	0.0682	0.0640
110011_ABEF	**0.0284**	42	0.0607	0.0763	0.0635	0.0703	0.0612
011011_BCEF	**0.0284**	42	0.0536	0.0763	0.0636	0.0751	0.0612
001011_CEF	**0.0260**	41	0.0743	0.0744	0.0568	0.0850	0.0594
100101_ADF	**0.0256**	44	0.0853	0.0768	0.0622	0.0791	0.0565
001101_CDF	**0.0256**	44	0.0853	0.0768	0.0622	0.0791	0.0565
010011_BEF	**0.0241**	41	0.0704	0.0753	0.0474	0.0864	0.0505
101111_ACDEF	**0.0198**	42	0.0733	0.0763	0.0510	0.0735	0.0494
101101_ACDF	**0.0188**	44	0.0701	0.0735	0.0511	0.0714	0.0491
010001_BF	**0.0187**	44	0.0716	0.0768	0.0487	0.0788	0.0525
011001_BCF	**0.0183**	43	0.0712	0.0735	0.0496	0.0787	0.0493
110001_ABF	**0.0183**	43	0.0720	0.0735	0.0508	0.0853	0.0493
011101_BCDF	**0.0152**	43	0.0738	0.0766	0.0432	0.0758	0.0350
110101_ABDF	**0.0152**	43	0.0729	0.0766	0.0436	0.0747	0.0350
101011_ACEF	**0.0142**	40	0.0731	0.0720	0.0464	0.0735	0.0441
010111_BDEF	**0.0142**	42	0.0780	0.0747	0.0489	0.0686	0.0474
010010_BE	**0.0130**	42	0.0301	0.0430	0.0535	0.0398	0.0509
111011_ABCEF	**0.0123**	43	0.0521	0.0754	0.0356	0.0781	0.0476
000010_E	**0.0111**	42	0.0427	0.0498	0.0434	0.0000	0.0487
000110_DE	**0.0099**	43	0.0252	0.0464	0.0488	0.0473	0.0413
101001_ACF	**0.0097**	38	0.0697	0.0757	0.0516	0.0802	0.0510
100110_ADE	**0.0093**	43	0.0048	0.0410	0.0515	0.0201	0.0548
001110_CDE	**0.0093**	43	0.0048	0.0410	0.0515	0.0201	0.0548
010110_BDE	**0.0092**	42	0.0044	0.0456	0.0457	0.0377	0.0517
110110_ABDE	**0.0079**	16	0.0024	0.0380	0.0298	0.0324	0.0211
011110_BCDE	**0.0079**	16	0.0024	0.0422	0.0245	0.0479	0.0231
010101_BDF	**0.0078**	40	0.0741	0.0745	0.0541	0.0743	0.0358
101110_ACDE	**0.0077**	16	-0.0003	0.0415	0.0299	0.0082	0.0307
100100_AD	**0.0074**	18	0.0650	0.0349	0.0388	0.0341	0.0339
001100_CD	**0.0074**	18	0.0650	0.0349	0.0388	0.0341	0.0339
001111_CDEF	**0.0074**	18	0.0726	0.0753	0.0457	0.0749	0.0376
100111_ADEF	**0.0074**	18	0.0726	0.0753	0.0460	0.0749	0.0376
111100_ABCD	**0.0073**	16	0.0478	0.0402	0.0511	0.0418	0.0507
111101_ABCDF	**0.0069**	15	0.0634	0.0775	0.0597	0.0600	0.0466
101010_ACE	**0.0068**	16	-0.0028	0.0444	0.0053	0.0444	0.0249
111010_ABCE	**0.0068**	16	-0.0113	0.0481	-0.0015	0.0104	-0.0133
110010_ABE	**0.0068**	16	0.0038	0.0459	0.0393	0.0182	0.0397
011010_BCE	**0.0068**	16	0.0561	0.0459	0.0357	0.0384	0.0327
111111_ABCDEF	**0.0068**	19	0.0573	0.0747	0.0379	0.0592	0.0293
111000_ABC	**0.0068**	16	0.0390	0.0331	0.0201	0.0324	0.0029
011000_BC	**0.0068**	16	0.0086	0.0357	0.0031	0.0358	-0.0027
110000_AB	**0.0068**	16	0.0283	0.0365	0.0034	0.0358	-0.0027
001010_CE	**0.0067**	18	0.0092	0.0470	0.0145	0.0487	-0.0011
100010_AE	**0.0067**	18	0.0092	0.0470	0.0145	0.0487	-0.0011
011100_BCD	**0.0066**	18	0.0454	0.0410	0.0193	0.0135	0.0178
110100_ABD	**0.0066**	18	0.0483	0.0332	0.0148	0.0036	0.0108
010000_B	**0.0065**	16	0.0291	0.0495	0.0278	0.0435	0.0351
101100_ACD	**0.0065**	16	0.0424	0.0368	0.0535	0.0396	0.0371
111110_ABCDE	**0.0064**	16	0.0181	0.0415	0.0461	-0.0042	0.0356
110111_ABDEF	**0.0064**	15	0.0722	0.0750	0.0341	0.0590	0.0282
011111_BCDEF	**0.0064**	15	0.0663	0.0750	0.0342	0.0509	0.0282
001000_C	**0.0058**	18	0.0325	0.0288	0.0400	0.0000	0.0364
100000_A	**0.0058**	18	0.0325	0.0288	0.0400	0.0000	0.0364
010100_BD	**0.0058**	18	0.0651	0.0519	0.0491	0.0368	0.0382
101000_AC	**0.0057**	16	0.0288	0.0400	0.0318	0.0327	0.0338

Table S4. MetaCAM HPC Results for Tie with Class 906 and ResNet152

Experiment ID	Max ROAD	Thresh. Max ROAD	MetaCAM Weighted	MetaCAM Softmax	MetaCAM Exp	MetaCAM Min-Max	MetaCAM
000111_DEF	**0.494**	28	0.371	0.383	0.387	0.340	0.382
000101_DF	**0.492**	35	0.375	0.385	0.392	0.378	0.379
001100_CD	**0.490**	29	0.373	0.388	0.371	0.387	0.374
100100_AD	**0.490**	29	0.373	0.388	0.371	0.387	0.374
010111_BDEF	**0.488**	25	0.372	0.380	0.391	0.365	0.394
001110_CDE	**0.486**	33	0.379	0.385	0.380	0.381	0.382
100110_ADE	**0.486**	33	0.379	0.385	0.380	0.381	0.382
101100_ACD	**0.486**	28	0.369	0.387	0.357	0.381	0.364
010101_BDF	**0.484**	26	0.385	0.390	0.388	0.375	0.386
010100_BD	**0.484**	33	0.370	0.389	0.383	0.378	0.378
000100_D	**0.484**	29	0.372	0.390	0.364	0.387	0.368
101101_ACDF	**0.483**	28	0.382	0.382	0.377	0.373	0.369
011100_BCD	**0.481**	29	0.367	0.387	0.369	0.379	0.364
110100_ABD	**0.481**	29	0.366	0.387	0.370	0.378	0.362
110101_ABDF	**0.480**	28	0.387	0.385	0.365	0.377	0.374
011101_BCDF	**0.480**	28	0.385	0.385	0.362	0.374	0.374
011110_BCDE	**0.478**	31	0.371	0.385	0.374	0.379	0.368
001101_CDF	**0.478**	40	0.390	0.385	0.375	0.372	0.369
100101_ADF	**0.478**	40	0.390	0.385	0.375	0.372	0.369
101110_ACDE	**0.478**	32	0.370	0.389	0.364	0.378	0.370
110110_ABDE	**0.476**	35	0.367	0.387	0.371	0.376	0.372
111100_ABCD	**0.473**	27	0.352	0.379	0.348	0.369	0.353
100111_ADEF	**0.472**	26	0.375	0.389	0.402	0.374	0.375
001111_CDEF	**0.472**	26	0.375	0.380	0.399	0.371	0.374
001011_CEF	**0.472**	34	0.301	0.277	0.357	0.287	0.364
100011_AEF	**0.472**	34	0.301	0.277	0.357	0.287	0.364
101111_ACDEF	**0.472**	37	0.387	0.387	0.374	0.366	0.367
001001_CF	**0.468**	36	0.371	0.297	0.362	0.297	0.345
010001_BF	**0.467**	34	0.345	0.294	0.372	0.265	0.366
110001_ABF	**0.467**	34	0.340	0.306	0.370	0.301	0.362
011001_BCF	**0.467**	34	0.341	0.304	0.367	0.294	0.364
100001_AF	**0.467**	36	0.365	0.306	0.362	0.295	0.349
101001_ACF	**0.465**	43	0.327	0.290	0.372	0.298	0.350
110111_ABDEF	**0.463**	28	0.377	0.389	0.391	0.376	0.366
011111_BCDEF	**0.463**	28	0.376	0.389	0.391	0.378	0.368
111110_ABCDE	**0.455**	28	0.368	0.386	0.363	0.377	0.345
111111_ABCDEF	**0.453**	28	0.374	0.386	0.363	0.372	0.353
000110_DE	**0.452**	44	0.337	0.384	0.343	0.383	0.335
101011_ACEF	**0.452**	44	0.320	0.297	0.337	0.268	0.340
010110_BDE	**0.451**	45	0.338	0.388	0.310	0.382	0.323
111101_ABCDF	**0.447**	42	0.379	0.388	0.344	0.369	0.339
111001_ABCF	**0.446**	43	0.294	0.286	0.359	0.269	0.345
010000_B	**0.443**	36	0.293	0.298	0.318	0.299	0.331
110011_ABEF	**0.438**	30	0.315	0.289	0.320	0.282	0.341
011011_BCEF	**0.438**	30	0.314	0.288	0.316	0.280	0.335
111000_ABC	**0.426**	29	0.275	0.299	0.297	0.297	0.308
010010_BE	**0.426**	39	0.300	0.300	0.305	0.263	0.310
100000_A	**0.418**	42	0.298	0.292	0.292	0.000	0.296
001000_C	**0.418**	42	0.298	0.292	0.292	0.000	0.296
101000_AC	**0.418**	34	0.292	0.292	0.297	0.297	0.301
111011_ABCEF	**0.410**	32	0.305	0.297	0.310	0.272	0.303
110000_AB	**0.404**	38	0.266	0.295	0.277	0.293	0.286
011000_BC	**0.404**	36	0.271	0.298	0.282	0.292	0.283
110010_ABE	**0.404**	45	0.282	0.291	0.292	0.269	0.302
011010_BCE	**0.404**	45	0.279	0.289	0.293	0.259	0.299
111010_ABCE	**0.397**	35	0.275	0.294	0.267	0.268	0.261
101010_ACE	**0.397**	37	0.270	0.291	0.269	0.298	0.274
100010_AE	**0.395**	37	0.282	0.276	0.289	0.292	0.288
001010_CE	**0.395**	37	0.282	0.276	0.289	0.292	0.288
010011_BEF	**0.379**	31	0.261	0.294	0.264	0.299	0.260
000011_EF	**0.371**	41	0.239	0.250	0.255	0.257	0.253
000010_E	**0.360**	44	0.256	0.262	0.260	0.000	0.255
000001_F	**0.148**	45	0.134	0.131	0.130	0.000	0.132

Table S5. MetaCAM HPC Results & Target Class Mismatch; Catdog Image with Dog Class 246 and DenseNet161

Experiment ID	Max ROAD	Thresh. Max ROAD	MetaCAM Weighted	MetaCAM Softmax	MetaCAM Exp	MetaCAM Min-Max	MetaCAM
001100_CD	**0.0117**	15	0.0004	0.0004	0.0003	0.0003	0.0003
010010_BE	**0.0008**	43	0.0003	0.0003	0.0003	0.0003	0.0003
011001_BCF	**0.0007**	15	0.0003	0.0003	0.0002	0.0003	0.0002
001010_CE	**0.0006**	25	0.0003	0.0004	0.0002	0.0003	0.0003
111100_ABCD	**0.0005**	20	0.0002	0.0005	0.0004	0.0003	0.0004
101111_ACDEF	**0.0005**	20	0.0003	0.0005	0.0004	0.0003	0.0004
111101_ABCDF	**0.0005**	19	0.0002	0.0005	0.0004	0.0003	0.0004
011101_BCDF	**0.0005**	19	0.0003	0.0005	0.0004	0.0003	0.0004
111111_ABCDEF	**0.0004**	20	0.0007	0.0005	0.0004	0.0003	0.0004
001101_CDF	**0.0004**	19	0.0004	0.0004	0.0004	0.0003	0.0004
101101_ACDF	**0.0004**	19	0.0003	0.0005	0.0003	0.0003	0.0004
101100_ACD	**0.0004**	18	0.0003	0.0005	0.0003	0.0003	0.0003
001111_CDEF	**0.0004**	18	0.0002	0.0005	0.0004	0.0002	0.0004
110010_ABE	**0.0004**	43	0.0003	0.0004	0.0003	0.0003	0.0003
001011_CEF	**0.0004**	32	0.0004	0.0003	0.0002	0.0003	0.0002
011111_BCDEF	**0.0004**	19	0.0004	0.0005	0.0004	0.0003	0.0003
001110_CDE	**0.0004**	44	0.0003	0.0005	0.0003	0.0003	0.0003
010110_BDE	**0.0004**	45	0.0003	0.0005	0.0004	0.0003	0.0004
001001_CF	**0.0004**	27	0.0004	0.0003	0.0003	0.0003	0.0003
011100_BCD	**0.0004**	18	0.0003	0.0005	0.0003	0.0003	0.0003
010000_B	**0.0004**	45	0.0003	0.0003	0.0004	0.0003	0.0004
101110_ACDE	**0.0004**	19	0.0003	0.0005	0.0003	0.0003	0.0003
111110_ABCDE	**0.0004**	20	0.0003	0.0004	0.0003	0.0003	0.0003
011010_BCE	**0.0004**	16	0.0003	0.0003	0.0003	0.0003	0.0003
101000_AC	**0.0003**	30	0.0004	0.0003	0.0003	0.0003	0.0003
110100_ABD	**0.0003**	45	0.0003	0.0005	0.0003	0.0003	0.0003
100010_AE	**0.0003**	42	0.0003	0.0003	0.0003	0.0003	0.0003
011011_BCEF	**0.0003**	19	0.0003	0.0003	0.0003	0.0004	0.0003
011110_BCDE	**0.0003**	19	0.0002	0.0005	0.0003	0.0003	0.0003
100011_AEF	**0.0003**	20	0.0003	0.0003	0.0003	0.0004	0.0003
110110_ABDE	**0.0003**	45	0.0003	0.0004	0.0003	0.0003	0.0003
110000_AB	**0.0003**	42	0.0003	0.0004	0.0003	0.0004	0.0003
101010_ACE	**0.0003**	42	0.0003	0.0003	0.0003	0.0003	0.0003
000010_E	**0.0003**	40	0.0004	0.0003	0.0004	0.0000	0.0003
010100_BD	**0.0003**	45	0.0003	0.0004	0.0003	0.0003	0.0003
111011_ABCEF	**0.0003**	18	0.0004	0.0003	0.0003	0.0004	0.0003
110101_ABDF	**0.0003**	45	0.0003	0.0005	0.0003	0.0003	0.0003
010011_BEF	**0.0003**	20	0.0004	0.0003	0.0004	0.0003	0.0004
110111_ABDEF	**0.0003**	45	0.0003	0.0005	0.0003	0.0003	0.0003
100100_AD	**0.0003**	45	0.0003	0.0004	0.0003	0.0003	0.0003
011000_BC	**0.0003**	44	0.0003	0.0003	0.0003	0.0004	0.0003
101011_ACEF	**0.0003**	41	0.0002	0.0003	0.0003	0.0003	0.0003
111010_ABCE	**0.0003**	21	0.0002	0.0003	0.0003	0.0003	0.0003
000011_EF	**0.0003**	20	0.0003	0.0004	0.0003	0.0003	0.0003
110011_ABEF	**0.0003**	38	0.0004	0.0004	0.0004	0.0003	0.0004
101001_ACF	**0.0002**	37	0.0003	0.0003	0.0002	0.0002	0.0002
100000_A	**0.0002**	42	0.0003	0.0003	0.0003	0.0000	0.0003
111001_ABCF	**0.0002**	16	0.0003	0.0004	0.0003	0.0003	0.0003
010101_BDF	**0.0002**	45	0.0003	0.0005	0.0003	0.0003	0.0003
111000_ABC	**0.0002**	45	0.0002	0.0003	0.0003	0.0003	0.0003
100101_ADF	**0.0002**	43	0.0003	0.0004	0.0003	0.0003	0.0003
000101_DF	**0.0002**	44	0.0003	0.0004	0.0003	0.0005	0.0003
110001_ABF	**0.0002**	42	0.0003	0.0003	0.0003	0.0003	0.0003
010001_BF	**0.0002**	37	0.0003	0.0003	0.0003	0.0003	0.0003
100110_ADE	**0.0002**	45	0.0003	0.0004	0.0003	0.0003	0.0003
100111_ADEF	**0.0002**	45	0.0002	0.0004	0.0003	0.0003	0.0003
000110_DE	**0.0002**	45	0.0003	0.0005	0.0003	0.0003	0.0003
010111_BDEF	**0.0002**	45	0.0003	0.0005	0.0003	0.0003	0.0003
100001_AF	**0.0002**	15	0.0002	0.0003	0.0002	0.0003	0.0002
000111_DEF	**0.0002**	31	0.0002	0.0004	0.0002	0.0003	0.0002
000001_F	**0.0002**	42	0.0003	0.0003	0.0003	0.0000	0.0003
000100_D	**0.0001**	27	0.0003	0.0004	0.0003	0.0005	0.0003

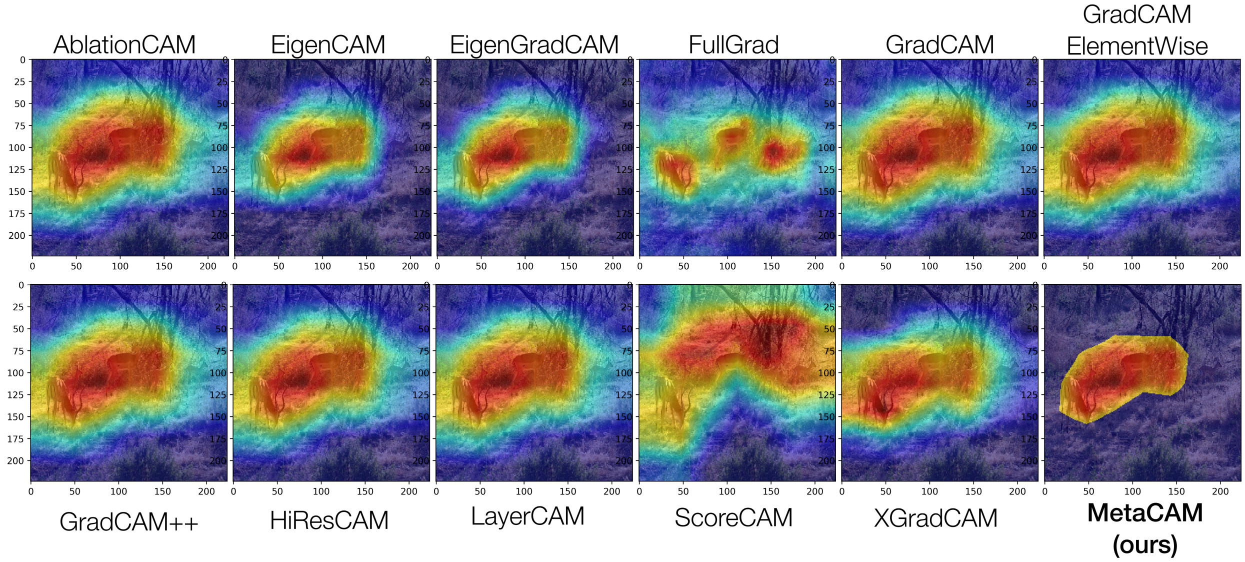

Figure S2. Comparison of MetaCAM to other CAM methods on "Grazing Impala" image.

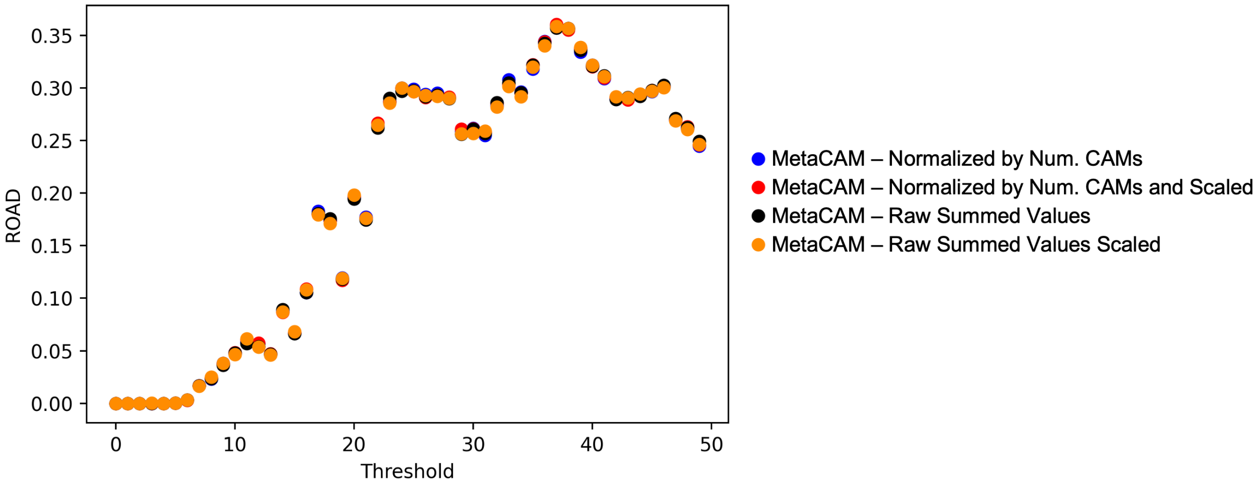

Figure S3. Effect of normalization and scaling on MetaCAM.

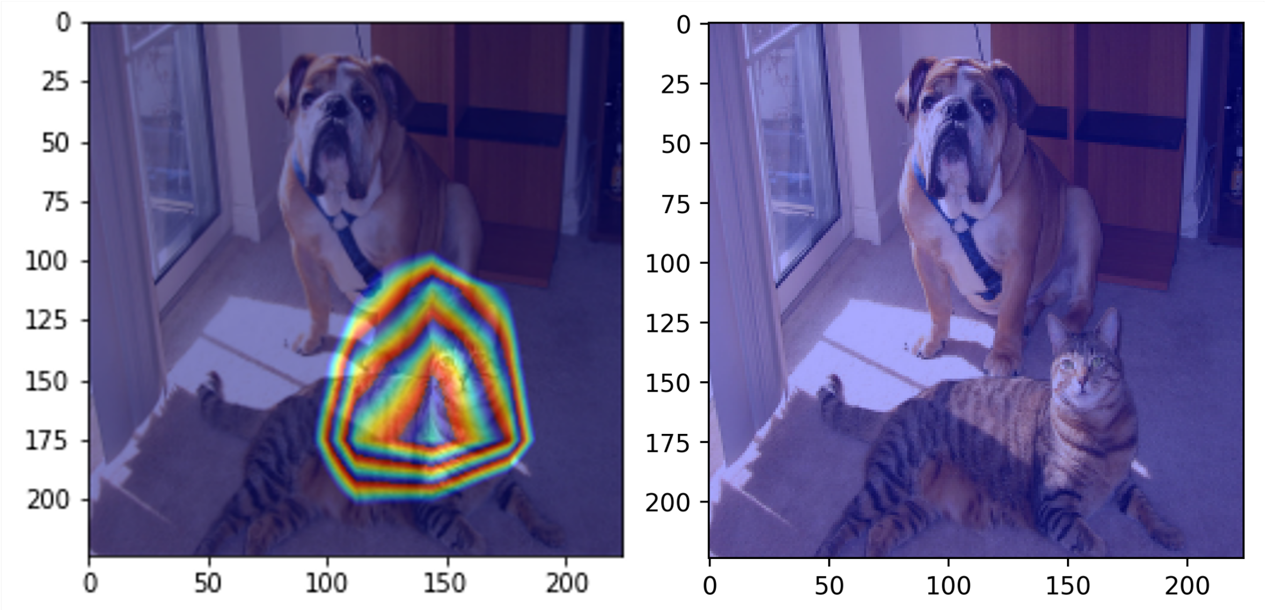

Figure S4. Example MetaCAM images outputted without scaling between [0,1].

6. Walker, M. C. *et al.* Using deep-learning in fetal ultrasound analysis for diagnosis of cystic hygroma in the first trimester. *Plos one* **17**, e0269323 (2022).

7. Al-Dhabyani, W., Gomaa, M., Khaled, H. & Fahmy, A. Dataset of breast ultrasound images. *Data brief* **28**, 104863 (2020).

8. Cardoso, M. J. *et al.* Monai: An open-source framework for deep learning in healthcare. *arXiv preprint arXiv:2211.02701* (2022).
